# Exploratory study of a meditation intervention program on Portuguese breast cancer survivors

**DOI:** 10.1192/j.eurpsy.2024.1338

**Published:** 2024-08-27

**Authors:** A. Torres, C. Ribeiro, S. R. Costa

**Affiliations:** ^1^Department of Psychology and Education, University of Beira Interior, Covilhã; ^2^Department of Education and Psychology of University of Aveiro, Center for Health Technology and Services Research (CINTESIS@RISE), Aveiro; ^3^Department of Education and Psychology, University of Trás-os-Montes e Alto Douro, Vila Real, Portugal

## Abstract

**Introduction:**

Cancer patients, namely breast cancer survivors, are highly vulnerable to psychological morbidity. Noninvasive interventions are incentivized to promote the mental health and quality of life of cancer survivors. Recent studies provided evidence supporting the use of meditation as a promising adjuvant tool for improving the mental health and quality of life of cancer survivors.

**Objectives:**

The present study aims to carry out a clinical trial to evaluate the effects of an online group of meditation program of Kundalini Yoga on breast cancer women, through a longitudinal and randomized research design, in the following variables: psychological morbidity, self-compassion, spirituality, and quality of life.

**Methods:**

This study had the participation of 35 participants distributed randomly for 3 equivalent groups (N=11 EG, N=13 ACG, N=11 PCG), with the diagnosis of breast cancer, aged between 34 and 78 years.

The sample of women with breast cancer was randomly selected from a breast cancer support association.

The protocol was applied online individually on pre-test, post-test, and 1-month follow-up moments, in 3 comparison groups: 1) the Experimental Group(EG), who practiced yoga Kundalini meditation; 2) the Active Control Group(ACG) that practiced relaxation; 3) the waiting list Passive Control Group(PCG). Intervention sessions were carried out for the EG and the ACG, in an online format, lasting about 30 minutes, weekly, for 8 weeks. Statistical analyses were considered at a 0.05 significance level. All analyses were performed with IBM SPSS, version 27.

**Results:**

The results showed that the group that did yoga kundalini meditation (EG) had benefits, unlike the control groups, in the variables of emotional functioning, global spiritual well-being, and personal well-being. There were statistically significant differences in the overall self-compassion score when comparing the 3-time points in all groups. The sub-scale of self-kindness and transcendental well-being shows an increase significantly between the 3 moments in the active control group. The passive control group performed significantly worse over time in the self-kindness.

**Conclusions:**

Based on preliminary results, the Experimental Group (EG) exhibited improvements in Self-Compassion, Spirituality, and Emotional Functioning (as evaluated by the QLQ C-30) following eight consecutive weeks of online Kundalini Yoga Meditation practice. These findings contribute to the growing body of evidence supporting meditation’s potential to enhance life quality and spiritual well-being in individuals with breast cancer. These preliminary findings suggest that further research in this promising field is warranted.

**Disclosure of Interest:**

None Declared

